# New Cembranoid Diterpenes from the Cultured Octocoral *Nephthea columnaris*

**DOI:** 10.3390/molecules200713205

**Published:** 2015-07-21

**Authors:** Ting-Hsi Hsiao, Ching-Hsiao Cheng, Tung-Ying Wu, Mei-Chin Lu, Wu-Fu Chen, Zhi-Hong Wen, Chang-Feng Dai, Yang-Chang Wu, Ping-Jyun Sung

**Affiliations:** 1 Graduate Institute of Marine Biology, National Dong Hwa University, Pingtung 94450, Taiwan; E-Mails: hsiaoinon@gmail.com (T.-H.H.); jinx6609@nmmba.gov.tw (M.-C.L.); 2 National Museum of Marine Biology & Aquarium, Pingtung 94450, Taiwan; 3 Department of Neurosurgery, Kaohsiung Chang Gung Memorial Hospital and Chang Gung University College of Medicine, Kaohsiung 83301, Taiwan; E-Mails: ma4200@cgmh.org.tw (C.-H.C.); ma4949@cgmh.org.tw (W.-F.C.); 4 Chinese Medicine Research and Development Center, China Medical University Hospital, Taichung 40447, Taiwan; E-Mail: kuma0401@gmail.com; 5 Department of Neurosurgery, Xiamen Chang Gung Memorial Hospital, Xiamen 361028, China; 6 Department of Marine Biotechnology and Resources, Asia-Pacific Ocean Research Center, National Sun Yat-sen University, Kaohsiung 80424, Taiwan; E-Mail: wzh@mail.nsysu.edu.tw; 7 Institute of Oceanography, National Taiwan University, Taipei 10617, Taiwan; E-Mail: corallab@ntu.edu.tw; 8 School of Pharmacy, College of Pharmacy, China Medical University, Taichung 40402, Taiwan; 9 Graduate Institute of Natural Products, Kaohsiung Medical University, Kaohsiung 80708, Taiwan; 10 Center for Molecular Medicine, China Medical University Hospital, Taichung 40447, Taiwan

**Keywords:** *Nephthea columnaris*, octocoral, cembrane, nephalsterol, cytotoxicity

## Abstract

Two new 15-hydroxycembranoid diterpenes, 2β-hydroxy-7β,8α-epoxynephthenol (**1**) and 2β-hydroxy-11α,12β-epoxynephthenol (**2**), were isolated from extracts of the octocoral *Nephthea columnaris* along with a new natural cembrane, epoxynephthenol (**3**) and a known sterol, nephalsterol A (**4**). The structures of cembranes **1**–**3** were elucidated by spectroscopic methods and comparison of the spectroscopic data with those of related analogues. The cytotoxicity of metabolites **1**–**4** against a panel of tumor cells is also described.

## 1. Introduction

**Figure 1:**
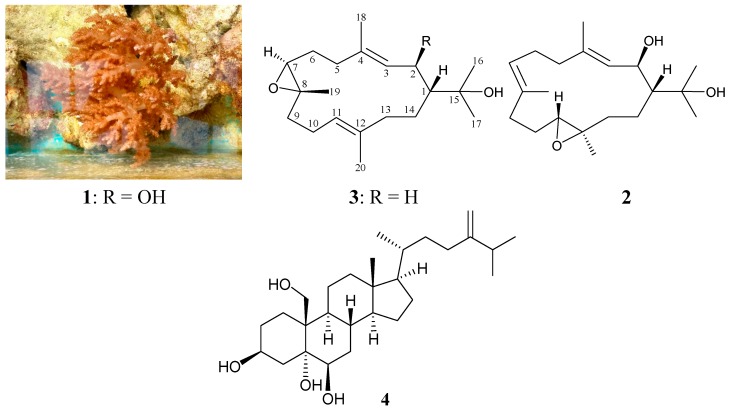
The soft coral *Nephthea columnaris* and the structures of 2β-hydroxy-7β,8α-epoxynephthenol (**1**), 2β-hydroxy-11α,12β-epoxynephthenol (**2**), epoxynephthenol (**3**) and nephalsterol A (**4**).

Previous chemical investigations of octocorals belonging to the genus *Nephthea*, collected off the waters of Taiwan, have yielded numbers of secondary metabolites, including steroids [1,2,3,4,5,6,7,8,9], sesquiterpenoids [3,5,6,10,11,12] and diterpenoids [10,13,14,15,16]. In our continuing studies, a sample collected off the coast of Southern Tip, Taiwan, identified as *Nephthea columnaris* (family Nephtheidae) (Figure 1) yielded three 15-hydroxycembranoid diterpenes, including two new compounds, 2β-hydroxy-7β,8α-epoxynephthenol (**1**) and 2β-hydroxy-11α,12β-epoxynephthenol (**2**), and a new natural cembrane, epoxynephthenol (**3**), along with a known sterol, nephalsterol A (**4**) [1,17] (Figure 1). In this paper, we report the isolation, structure determination and cytotoxicity of compounds **1**–**4**.

## 2. Results and Discussion

2β-Hydroxy-7β,8α-epoxynephthenol (**1**) was isolated as a colorless oil. The molecular formula for this compound was determined to be C_20_H_34_O_3_ (corresponding to four unsaturations) using HRESIMS (C_20_H_34_O_3_ + Na, *m*/*z* 345.23989, calcd. 345.24002). Comparison of the ^13^C-NMR and DEPT data with the molecular formula indicated there must be two exchangeable protons, which required the presence of two hydroxy groups. This deduction was supported by a broad absorption at 3361 cm^–1^ in the IR spectrum. The ^13^C-NMR data for **1** confirmed the presence of twenty carbon signals (Table 1), characterized by DEPT spectrum as five methyls, six sp^3^ methylenes, three sp^3^ methines (including two oxymethines), two sp^3^ oxygenated quaternary carbons, two sp^2^ methines and two sp^2^ quaternary carbons. Based on the ^1^H- and ^13^C-NMR spectra (Table 1), **1** was determined to contain two isolated methyl-bearing trisubstituted double bonds. The presence of a trisubstituted epoxide containing a methyl substituent was established from the signals of an oxygenated quaternary carbon (δ_C_ 60.2, C-8) and an oxymethine (δ_C_ 62.3; δ_H_ 2.61, 1H, dd, *J* = 6.0, 5.6 Hz, CH-7) and further confirmed by the proton signal of a methyl singlet at δ_H_ 1.28 (3H, s, H_3_-19). Thus, from the reported data, the proposed skeleton of **1** was suggested to be a cembrane-type diterpene with two rings.

**Table 1:** ^1^H (400 MHz, CDCl_3_) and ^13^C (100 MHz, CDCl_3_) NMR data, ^1^H-^1^H COSY and HMBC correlations for cembrane **1**.

Position	δ_H_ (*J* in Hz)	δ_C_, Multiple	^1^H-^1^H COSY	HMBC
1	1.49 m	54.9, CH	H-2, H_2_-14	C-2, -15
2	4.51 dd (10.0, 9.6)	71.4, CH	H-1, H-3	C-1, -3, -4, -15
3	5.40 dd (9.6, 1.2)	127.3, CH	H-2, H_3_-18	C-5, -18
4		136.2, C		
5	2.35–2.17 m	34.5, CH_2_	H_2_-6	C-3, -4, -6, -7, -18
6	1.78–1.69 m	27.1, CH_2_	H_2_-5, H-7	C-4, -5, -7, -8
7	2.61 dd (6.0, 5.6)	62.3, CH	H_2_-6	C-5, -6, -8, -9
8		60.2, C		
9	2.07 ddd (13.2, 4.8, 3.6); 1.08 m	39.1, CH_2_	H_2_-10	C-7, -8, -10, -11, -19
10	2.22 m; 1.93 m	23.3, CH_2_	H_2_-9, H-11	C-9
11	5.14 dd (8.4, 7.6)	123.3, CH	H_2_-10, H_3_-20	C-10, -13, -20
12		139.7, C		
13	2.17 m; 1.96 m	42.5, CH_2_	H_2_-14	C-11, -12, -14
14	1.29 m; 0.80 dddd (14.4, 11.6, 3.2, 3.2)	28.6, CH_2_	H-1, H_2_-13	C-1, -2, -13, -15
15		75.1, C		
16	1.28 s	30.1, CH_3_		C-1, -15, -17
17	1.28 s	23.9, CH_3_		C-1, -15, -16
18	1.84 d (1.2)	17.9, CH_3_	H-3	C-3, -4, -5
19	1.28 s	16.2, CH_3_		C-7, -8, -9
20	1.53 s	15.4, CH_3_	H-11	C-11, -12, -13

From the ^1^H-^1^H COSY spectrum of **1** (Table 1), it was possible to differentiate among the separate H-3/H-2/H-1/H_2_-14/H_2_-13, H_2_-5/H_2_-6/H-7 and H_2_-9/H_2_-10/H-11 spin systems. These data, together with the HMBC correlations between H-1/C-2; H-2/C-1, -3, -4; H-3/C-5; H_2_-5/C-3, -4, -6, -7; H_2_-6/C-4, -5, -7, -8; H-7/C-5, -6, -8, -9; H_2_-9/C-7, -8, -10, -11; H_2_-10/C-9; H-11/C-10, -13; H_2_-13/C-11, -12, -14; and H_2_-14/C-1, -2, -13, observed in an HMBC experiment, established the connectivity from C-1 to C-14 in a 14-membered ring. The vinyl methyls attached at C-4 and C-12 were confirmed by the HMBC correlations between H-3, H_2_-5/C-18; H_3_-18/C-3, -4, -5; and H-11/C-20; H_3_-20/C-11, -12, -13, and were further supported by the allylic couplings between H-3/H_3_-18 and H-11/H_3_-20. An isopropyl carbinol group at C-1 was elucidated by the HMBC correlations between H_3_-16/C-1, -15, -17; H_3_-17/C-1, -15, -16; and H-1, H-2, H_2_-14/C-15. The C-7/8 epoxide group was confirmed by the HMBC correlations between H_2_-5, H_2_-6, H_2_-9/C-7; H_2_-6, H-7, H_2_-9/C-8; and H_3_-19/C-7, -8, -9. Thus, the remaining hydroxy group was positioned at C-2, an oxygen-bearing methine (δ_H_ 4.51, 1H, dd, *J* = 10.0, 9.6 Hz; δ_C_ 71.4, CH-2). Based on the above findings, the planar structure of **1** was established.

**Figure 2:**
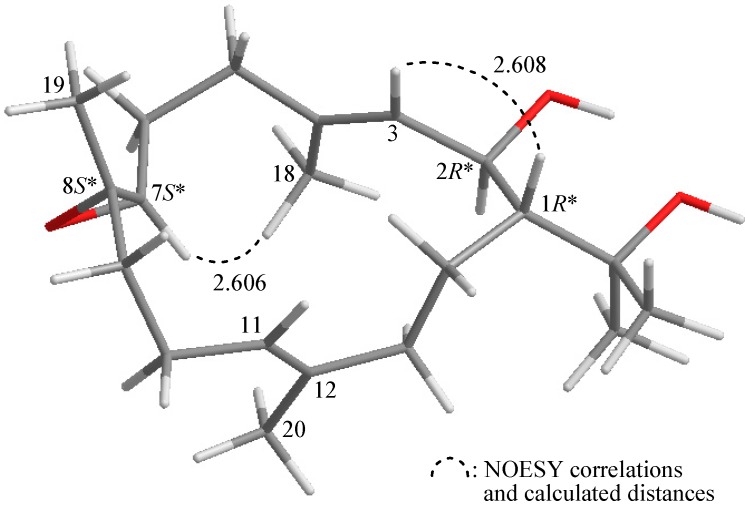
The computer-generated model of **1** using MM2 force field calculations and the calculated distance (Å) between selected protons with key NOESY correlations.

The relative configuration of **1** was elucidated mainly from a NOESY spectrum as being compatible with that of **1** ascertained using molecular mechanics calculations (MM2) [18], which suggested the most stable conformation to be as shown in Figure 2, in which the calculated close contacts of atoms in space were consistent with the NOESY correlations. The β-orientation of H-1, its NOESY correlation with H-3, but not with H-2, and the existence of large coupling constants between H-1/H-2 (*J* = 10.0 Hz) and H-2/H-3 (*J* = 9.6 Hz), indicated that the dihedral angles between H-1/H-2 and H-2/H-3 are approximately 180° [19] and the configurations of both chiral carbons C-1 and C-2 were assigned as R*-form. No correlation was found between H-3/H_3_-18 and H-11/H_3_-20, indicating that C-3/4 and C-11/12 carbon-carbon double bonds had an *E*-configuration. An NOESY interaction could be observed between H-7 and H_3_-18, but H-7 did not correlate with H_3_-19, revealing that H-7 and Me-19 should be α- and β-oriented, respectively. Furthermore, comparison of the NMR chemical shifts and coupling pattern of CH-7 (δ_H_ 2.61, 1H, dd, *J* = 6.0, 5.6 Hz; δ_C_ 62.3) and C-8 (δ_C_ 60.2) in **1** with those of a known cembrane analogue, (2*R*,7*S*,8*S*)-sarcophytoxide (δ_H_2.60, 1H, t, *J* = 5.6 Hz; δ_C_ 62.3, CH-7; δ_C_ 60.1, C-8) [20], indicated that the chiral carbons C-7 and C-8 possessed S*-configuration.

2β-Hydroxy-11α,12β-epoxynephthenol (**2**) had the same molecular formula as that of **1**, C_20_H_34_O_3_, with four degrees of unsaturation, as determined by HRESIMS. The spectroscopic data (IR, MS, ^1^H- and ^13^C-NMR) of **2** were very similar to those of **1**. However, the optical rotation of **2** ${\text{[α]}}_{D}^{24}$ –3, *c* 0.5, MeOH) was substantially different from that of **1** (${\text{[α]}}_{D}^{23}$ –43, *c* 0.8, MeOH), indicating that these two compounds are isomers. The NMR chemical shifts for 7,8-epoxy (δ_H_ 2.61, 1H, dd, *J* = 6.0, 5.6 Hz, H-7; δ_C_ 62.3, CH-7; 60.2, C-8) and 11,12-olefinic (δ_H_ 5.14, 1H, dd, *J* = 8.4, 7.6 Hz, H-11; δ_C_ 123.3, CH-11; 139.7, C-12) groups in **1** were found to be shifted by comparison of these data with those of **2** (δ_H_ 5.02, 1H, dd, *J* = 8.0, 6.4 Hz, H-7; δ_C_ 124.7, CH-7; 133.0, C-8; δ_H_ 2.67, 1H, dd, *J* = 9.6, 2.8 Hz, H-11; δ_C_ 62.8, CH-11; 61.1, C-12) (Table 2), indicating that the 7,8-epoxy and 11,12-olefinic groups in compound **1** were replaced by olefinic and epoxy groups, respectively, in **2**. The 2D NMR correlations observed fully supported the locations of functional groups (Table 2). On the basis of the above observations, the planar structure of cembrane **2** was established. 

**Table 2:** ^1^H (400 MHz, CDCl_3_) and ^13^C (100 MHz, CDCl_3_) NMR Data, ^1^H-^1^H COSY and HMBC correlations for cembrane **2**.

Position	δ_H_ (*J* in Hz)	δ_C_, Multiple	^1^H-^1^H COSY	HMBC
1	1.38 ddd (10.0, 4.8, 2.0)	54.4, CH	H-2, H_2_-14	C-2, -3, -13, -14, -15, -16, -17
2	4.46 dd (10.0, 8.8)	71.0, CH	H-1, H-3	C-1, -3, -4, -15
3	5.18 d (8.8)	127.9, CH	H-2, H_3_-18	C-1, -5, -18
4		140.8, C		
5	2.33 m; 1.95 m	39.9, CH_2_	H_2_-6	C-3, -4, -6, -7, -18
6	2.31 m; 2.18 m	24.2, CH_2_	H_2_-5, H-7	C-5, -7, -8
7	5.02 dd (8.0, 6.4)	124.7, CH	H_2_-6, H_3_-19	C-6, -9, -19
8		133.0, C		
9	2.20 m; 1.98 m	36.8, CH_2_	H_2_-10	C-7, -8, -10, -19
10	2.00 m; 1.28 m	24.4, CH_2_	H_2_-9, H-11	C-8, -9, -11, -12
11	2.67 dd (9.6, 2.8)	62.8, CH	H_2_-10	C-10
12		61.1, C		
13	2.16 m; 0.99 m	42.3, CH_2_	H_2_-14	C-1, -11, -12, -14, -20
14	1.12–0.95 m	26.1, CH_2_	H-1, H_2_-13	C-1, -2, -13, -15
15		74.7, C		
16	1.26 s	29.9, CH_3_		C-1, -15, -17
17	1.30 s	24.0, CH_3_		C-1, -15, -16
18	1.77 s	15.2, CH_3_	H-3	C-3, -4, -5
19	1.64 s	14.9, CH_3_	H-7	C-7, -8, -9
20	1.18 s	15.8, CH_3_		C-11, -12, -13

In order to deduce the relative stereochemistry at C-1/C-2/C-3 fragments, proton-proton coupling constant analysis revealed the same stereochemistry as in **1**. This was confirmed from the NOESY correlation between H-1 and H-3 and large coupling constants were recorded between H-1/H-2 (*J* = 10.0 Hz) and H-2/H-3 (*J* = 8.8 Hz) and the configurations of both chiral carbons C-1 and C-2 were assigned as R*-form. No correlation was found between H-3/H_3_-18 and H-7/H_3_-19, indicating that C-3/4 and C-7/8 carbon-carbon double bonds had an *E*-configuration. One proton attaching at C-13 and resonating at δ_H_ 0.99 was found to show interactions with H-1 and H-11 and was assigned arbitrary as H-13β. The other proton attaching at C-13, H-13α, showed interaction with H_3_-20, but not with H-1, confirming that H-11 and Me-20 should be β- and α-oriented, respectively. Moreover, comparison of the NMR chemical shifts and coupling pattern of CH-11 (δ_H_ 2.67, 1H, dd, *J* = 9.6, 2.8 Hz; δ_C_ 62.8) and C-12 (δ_C_ 61.1) in **2** with those of a known cembrane analogue, sinugibberol (δ_H_ 2.69, 1H, dd, *J* = 10.4, 2.9 Hz; δ_C_ 62.1, CH-11; δ_C_ 61.2, C-12) [21], indicated that the chiral carbons C-11 and C-12 possessed R*-configuration.

Cembrane **3**, obtained as a colorless oil, showed an [M + Na]^+^ signal at *m*/*z* 329.24507 in the HRESIMS, suggesting the molecular formula C_20_H_34_O_2_ (calcd. C_20_H_34_O_2_ + Na, 329.24510), with four degrees of unsaturation. The IR absorption of **3** at 3421 cm^–1^ indicated the presence of hydroxy functionality. It was found that the ^1^H- and ^13^C-NMR data of **3** (Table 3) were similar to those of **1**, except that the signals corresponding to the C-2, a hydroxy-bearing oxymethine group in **1** (δ_H_ 4.51, 1H, dd, *J* = 10.0, 9.6 Hz; δ_C_ 71.4, CH-2) were replaced by a methylene group in **3** (δ_H_ 2.32, 1H, m; 1.85, 1H, ddd, *J* = 14.0, 8.0, 6.4 Hz; δ_C_ 28.9, CH_2_-2). In the NOESY experiment of **3**, H-3 correlated with H-1, H-7 and H-11, indicating that the configurations of chiral carbons C-1 and C-7 should be assigned as R*- and S*-forms, respectively. No correlation was found between H-3/H_3_-18 and H-11/H_3_-20, indicating that C-3/4 and C-11/12 carbon-carbon double bonds had an *E*-configuration. Comparison of the NMR chemical shifts and coupling pattern of CH-7 (δ_H_ 2.86, dd, *J* = 5.6, 4.8 Hz; δ_C_ 62.2) and C-8 (δ_C_ 59.9) in **3** with those of a known cembrane analogue, epoxynephthenol acetate (δ_C_ 62.3, CH-7; δ_C_ 60.0, C-8) [22], (δ_H_ 2.87, t, *J* = 5.3 Hz; δ_C_ 62.2, CH-7; δ_C_ 59.9, C-8) [23], (δ_C_ 62.1, CH-7; δ_C_ 59.7, C-8) [24], indicating the chiral carbons C-7 and C-8 possessed S*-configuration. In a previous study, compound **3** as presented here had been obtained by reduction of epoxynephthenol acetate and named as epoxynephthenol [25]. To the best of our knowledge, this is the first time that cembrane **3** has been obtained from a natural source. The spectroscopic data of **3** were also reported in this study.

**Table 3:** ^1^H (400 MHz, CDCl_3_) and ^13^C (100 MHz, CDCl_3_) NMR data, ^1^H-^1^H COSY and HMBC correlations for cembrane **3**.

Position	δ_H_ (*J* in Hz)	δ_C_, Multiple	^1^H-^1^H COSY	HMBC
1	1.26 m	48.0, CH	H_2_-2, H_2_-14	C-2, -15
2	2.32 m; 1.85 ddd (14.0, 8.0, 6.4)	28.9, CH_2_	H-1, H-3	C-3, -4, -14, -15
3	5.34 ddq (8.0, 8.0, 1.2)	127.0, CH	H_2_-2, H_3_-18	C-2, -5, -18
4		131.7, C		
5	2.28 m; 2.19 m	36.4, CH_2_	H_2_-6	C-3, -4, -6, -7, -18
6	1.78–1.65 m	25.3, CH_2_	H_2_-5, H-7	C-5, -7, -8
7	2.86 dd (5.6, 4.8)	62.2, CH	H_2_-6	C-5, -6, -8, -9
8		59.9, C		
9	2.04 ddd (13.2, 6.4, 2.8); 1.23–1.17 m	38.7, CH_2_	H_2_-10	C-7, -8, -10, -11
10	2.23 m; 1.94 m	23.3, CH_2_	H_2_-9, H-11	C-8, -9, -11
11	5.09 ddq (7.2, 7.2, 1.2)	124.9, CH	H_2_-10, H_3_-20	C-10, -13, -20
12		135.0, C		
13	2.18–2.12 m	36.4, CH_2_	H_2_-14	C-1, -11, -12, -14, -20
14	1.73 m; 1.34 m	28.8, CH_2_	H_2_-13, H-1	C-1, -2, -12, -13, -15
15		73.7, C		
16	1.20 s	27.8, CH_3_		C-1, -15, -17
17	1.21 s	27.0, CH_3_		C-1, -15, -16
18	1.67 br s	15.7, CH_3_	H-3	C-3, -4, -5
19	1.29 s	17.0, CH_3_		C-7, -8, -9
20	1.55 br s	15.1, CH_3_	H-11	C-11, -12, -13

A known sterol, nephalsterol A (= 24-methylcholesta-24(28)-ene-3β,5α,6β,19-tetraol) (**4**), was also obtained in this study. This compound had been previously isolated from the soft corals *Nephthea erecta* [1] and *Nephthea albida* [17], respectively. The ^1^H- and ^13^C-NMR data of **4** were identical to those of known sterols described previously [1,17], confirming that **4** was nephalsterol A.

The cytotoxicity of compounds **1**–**4** against the proliferation of a limited panel of tumor cell lines, including MOLT-4 (human acute lymphoblastic leukemia), SUP-T1 (human T-cell lymphoblastic lymphoma), U-937 (human histiocytic lymphoma), DLD-1 (human colorectal adenocarcinoma), LNCaP (human prostatic carcinoma) and MCF7 (human breast adenocarcinoma) was studied. The results showed that compounds **1**−**3** are not cytotoxic toward the above cells (IC_50_ > 20 μg/mL). Sterol **4** was found to exhibit cytotoxicity toward above cells (IC_50_ = 22.5, 32.4, 38.6, 44.2, 11.6 and 9.8 μM). The anticancer agent doxorubicin was used as the positive control and exhibited IC_50_ values of 0.04, 0.09, 0.42, 0.20, 0.35 and 1.99 μM against MOLT-4, SUP-T1, U-937, DLD-1, LNCaP and MCF7 cells, respectively.

## 3. Experimental Section

### 3.1. General Procedures

Optical rotation values were measured with a Jasco P-1010 digital polarimeter (Japan Spectroscopic Corporation, Tokyo, Japan). IR spectra were obtained on a Varian Digilab FTS 1000 FT-IR spectrophotometer (Varian Inc., Palo Alto, CA, USA); absorptions are reported in cm^−^^1^. NMR spectra were recorded on a Varian Mercury Plus 400 NMR spectrometer using the residual solvent signals (CDCl_3_, δ_H_ 7.26 ppm for ^1^H-NMR and δ_C_ 77.1 ppm for ^13^C-NMR) as the internal standard. Coupling constants (*J*) are given in Hz. ESIMS and HRESIMS were recorded using a Bruker 7 Tesla solariX FTMS system (Bruker, Bremen, Germany). Column chromatography was performed on silica gel (230–400 mesh, Merck, Darmstadt, Germany). TLC was carried out on precoated Kieselgel 60 F_254_ (0.25 mm, Merck); spots were visualized by spraying with 10% H_2_SO_4_ solution followed by heating. Normal-phase HPLC (NP-HPLC) was performed using a system comprised of a Hitachi L-7110 pump (Hitachi Ltd., Tokyo, Japan), a Hitachi L-7455 photodiode array detector and a Rheodyne 7725 injection port (Rheodyne LLC, Rohnert Park, CA, USA). A normal-phase column (Supelco Ascentis^®^ Si Cat #: 581515-U, 25 cm × 21.2 mm, 5 μm, Sigma-Aldrich Co., St. Louis, MO, USA) was used for HPLC. The reversed phase HPLC (RP-HPLC) was performed using a system comprised of a Hitachi L-2130 pump, a Hitachi L-2455 photodiode array detector and a Rheodyne 7725 injection port. A reversed phase column (Supelco Ascentis^®^ Si Cat #: 581343-U, 25 cm × 10.0 mm, 5 μm, Sigma-Aldrich Co.) was used for RP-HPLC.

### 3.2. Animal Material

Specimens of the octocoral *Nephthea columnaris* (Studer, 1895) were collected by hand using SCUBA equipment off the coast of the Southern Taiwan, and transplanted to five 0.6-ton cultivating tanks equipped with a flow-through sea water system in February 2012. The cultured octocorals for this research work were collected from the tanks in May 2013. Living reference specimen are being maintained in the authors’ marine organism cultivating tank and a voucher specimen (NMMBA-TWSC-12005) was deposited in the National Museum of Marine Biology and Aquarium, Taiwan.

### 3.3. Extraction and Isolation

Sliced bodies of *Nephthea columnaris* (wet weight 800.0 g, dry weight 76.6 g) were extracted with a mixture of methanol (MeOH) and dichloromethane (DCM) (1:1, 1.6 L × 5). The extract was partitioned between ethyl acetate (EtOAc) and water (1:1, 0.8 L × 6). The concentrated EtOAc layer (7.4 g) was separated on silica gel and eluted using *n*-hexane/EtOAc (stepwise, 100:1–pure EtOAc) to yield 17 fractions A–Q. Fraction J was chromatographed on NP-HPLC using a mixture of *n*-hexane and acetone (2:1) to afford 14 fractions J1–J14. Fraction J3 was separated by NP-HPLC using a mixture of *n*-hexane and acetone (2:1) as the mobile phase to yield six fractions J3A–J3F. Fraction J3B was purified by RP-HPLC using a mixture of acetonitrile and water (1:1, flow rate: 2.0 mL/min) to afford **1** (2.3 mg, *t*_R_ = 16 min) and **2** (1.4 mg, *t*_R_ = 55 min). Fraction F was chromatographed on NP-HPLC using a mixture of *n*-hexane and acetone (4:1) to afford eight fractions F1–F8. Fraction F3 was separated by RP-HPLC using a mixture of methanol and water (17:3, flow rate: 1.0 mL/min) to afford **3** (1.8 mg, *t*_R_ = 27 min). Fraction L was chromatographed on NP-HPLC using a mixture of *n*-hexane and acetone (5:2) to afford 16 fractions L1–L16. Fraction L9 was separated by NP-HPLC using a mixture of DCM and acetone (5:1, flow rate: 3.0 mL/min) as the mobile phase to afford **4** (1.9 mg, *t*_R_ = 122 min).

*2β-Hydroxy-7β,8α-epoxynephthenol* (**1**): colorless oil; ${\text{[α]}}_{D}^{23}$ –43 (*c* 0.77, MeOH); IR (neat) ν_max_ 3361 cm^−1^; ^1^H (400 MHz, CDCl_3_) and ^13^C (100 MHz, CDCl_3_) NMR data, see Table 1; ESIMS: *m*/*z* 345 [M + Na]^+^; HRESIMS: *m*/*z* 345.23989 (calcd for C_20_H_34_O_3_ + Na, 345.24002).

*2β-Hydroxy-11α,12β-epoxynephthenol* (**2**): colorless oil; ${\text{[α]}}_{D}^{24}$ –3 (*c* 0.52, MeOH); IR (neat) ν_max_ 3419 cm^−1^; ^1^H (400 MHz, CDCl_3_) and ^13^C (100 MHz, CDCl_3_) NMR data, see Table 2; ESIMS: *m*/*z* 345 [M + Na]^+^; HRESIMS: *m*/*z* 345.23987 (calcd for C_20_H_34_O_3_ + Na, 345.24002).

*Epoxynephthenol* (**3**): colorless oil; ${\text{[α]}}_{D}^{24}$ –63 (*c* 0.60, MeOH); IR (neat) ν_max_ 3421 cm^−1^; ^1^H (400 MHz, CDCl_3_) and ^13^C (100 MHz, CDCl_3_) NMR data, see Table 3; ESIMS: *m*/*z* 329 [M + Na]^+^; HRESIMS: *m*/*z* 329.24507 (calcd for C_20_H_34_O_2_ + Na, 329.24510).

*Nephalsterol A* (**4**): white solid; ${\text{[α]}}_{D}^{25}$ +11 (*c* 0.63, MeOH) (ref. [1] ${\text{[α]}}_{D}^{25}$ +2.8 (*c* 0.12, MeOH); ref. [16] ${\text{[α]}}_{D}^{24}$ +2.6 (*c* 0.347, MeOH)); IR (neat) ν_max_ 3352 cm^−1^; The ^1^H (400 MHz, DMSO-*d*_6_) and ^13^C (100 MHz, DMSO-*d*_6_) NMR data of **4** were in full agreement with those reported previously [1,17]; ESIMS: *m*/*z* 471 [M + Na]^+^.

### 3.4. Molecular Mechanics Calculations

Implementation of the MM2 force field [18] in CHEM3D PRO software from CambridgeSoft Corporation (Cambridge, MA, USA; ver. 9.0, 2005) was used to calculate molecular models.

### 3.5. MTT Antiproliferative Assay

MOLT-4, SUP-T1, U-937, DLD-1, LNCaP and MCF7 cells were obtained from the American Type Culture Collection (ATCC, Manassas, VA, USA). Cells were maintained in RPMI 1640 medium supplemented with 10% fetal calf serum, 2 mM glutamine and antibiotics (100 units/mL penicillin and 100 μg/mL streptomycin) at 37 °C in a humidified atmosphere of 5% CO_2_. Cells were seeded at 4 × 10^4^ per well in 96-well culture plates before treatment with different concentrations of the tested compounds. The compounds were dissolved in dimethyl sulfoxide (less than 0.02%) and made concentrations of 1.25, 2.5, 5, 10 and 20 μg/μL prior to the experiments. After treatment for 72 h, the cytotoxicity of the tested compounds was determined using a MTT cell proliferation assay (thiazolyl blue tetrazolium bromide, Sigma-M2128). The MTT is reduced by the mitochondrial dehydrogenases of viable cells to a purple formazan product. The MTT-formazan product was dissolved in DMSO. Light absorbance values (OD = OD_570_ − OD_620_) were recorded at wavelengths of 570 and 620 nm using an ELISA reader (Anthos labtec Instrument, Salzburg, Austria) to calculate the concentration that caused 50% inhibition (IC_50_), *i.e.*, the cell concentration at which the light absorbance value of the experiment group was half that of the control group. These results were expressed a percentage of the control ± SD established from *n* = 4 wells per one experiment from three separate experiments [26,27,28].

## 4. Conclusions

A series of cembrane-based diterpenoid and steroid metabolites were isolated from soft corals belonging to the genus *Nephthea*, collected off the waters of Taiwan. Our continued investigation on the chemical constituents of *N. columnaris* has led to the isolation of two new cembranoids, 2β-hydroxy-7β,8α-epoxynephthenol (**1**) and 2β-hydroxy-11α,12β-epoxynephthenol (**2**), a new natural cembrane, epoxynephthenol (**3**) and a known sterol, nephalsterol A (**4**). To the best of our knowledge, this is the first time to study the natural products from *N. columnaris*. Nephasterol A (**4**) exhibited moderate cytotoxicity against MOLT-4, SUP-T1, U-937, DLD-1, LNCaP and MCF7 cells.
